# A cost-effectiveness study of ICT training among the visually impaired in the Netherlands

**DOI:** 10.1186/s12886-018-0761-y

**Published:** 2018-04-17

**Authors:** Nathalie J. S. Patty, Marc Koopmanschap, Kim Holtzer-Goor

**Affiliations:** Erasmus School of Health Policy & Management, Erasmus University, P.O. Box 1738, 3000 DR Rotterdam, The Netherlands

**Keywords:** Cost-effectiveness, ICECAP-O, Visually impaired, Eye care, Rehabilitation, ICT training

## Abstract

**Background:**

Due to the ageing population, the number of visually impaired people in the Netherlands will increase. To ensure the future availability of services in rehabilitative eye care, we aim to assess the cost-effectiveness of information and communication technology (ICT) training among visually impaired adults from a societal perspective, using primary data from two large rehabilitative eye care providers in the Netherlands.

**Methods:**

Participants were asked to fill in a questionnaire, which used six different instruments at three different time points: pre training, post training and three months post training. We investigated whether the participants’ quality of life and well-being improved after the training and whether this improvement persisted three months post training. Economic evaluation was conducted by comparing costs and outcomes before and after training. Quality of life and well-being were derived from the EQ-5D and ICECAP-O, respectively. Costs for productivity losses and medical consumption were obtained from the questionnaires. Information regarding the costs of training sessions was provided by the providers.

**Results:**

Thirty-eight participants filled in all three questionnaires. The mean age at baseline was 63 years (SD = 16). The effect of ICT training on ICT skills and participants’ well-being was positive and persisted three months after the last training session. Assuming these effects remain constant for 10 years, this would result in an incremental cost-effectiveness ratio (ICER) of € 11,000 per quality-adjusted life-year (QALY) and € 8000 per year of well-being gained, when only the costs of ICT training are considered. When the total costs of medical consumption are included, the ICER increases to € 17,000 per QALY gained and € 12,000 per year of well-being gained. Furthermore, when the willingness-to-pay threshold is € 20,000 per year of well-being, the probability that ICT training will be cost-effective is 75% (91% when including only the costs of ICT training).

**Conclusion:**

Our study suggests that ICT training among the visually impaired is cost-effective when the effects of ICT training on well-being persist for several years. However, further research involving a larger sample and incorporating long-term effects should be conducted.

## Background

In the Netherlands, approximately 320,000 people live with a visual impairment of both eyes (visual acuity of < 0.3 and logMAR approximately < 0.5 with applicable corrections); of these, approximately 45,000 are considered blind (visual acuity of < 0.05 and logMAR < 1.3). The probability of becoming visually impaired increases with age, and 85% of all visually impaired people in the Netherlands are 50 years or older. It has been estimated that due to the ageing population, the number of visually impaired people in the Netherlands will increase to approximately 400,000 in 2020 if eye care remains at its current standard [1, 2].

Social health insurance in the Netherlands financially covers assistance and rehabilitative care for people with visual impairments as a part of standard care. These rehabilitative services include support and counseling, with the objective of enabling people to live as independently as possible. As people with visual impairments face distinct barriers in relation to information and communication technology (ICT) tools [2], one of the rehabilitative services covered is ICT training. ICT skills are essential for social interactions and information searching and hence necessary for full participation in society [2]. To maintain financial coverage for such rehabilitative care services, it is important to understand what the health and/or well-being gains are in relation to the financial investments for these services. Cost-effectiveness analysis (CEA) aims to assess the costs and gains of healthcare policies, services or interventions and hence inform decision-makers about the benefits and costs of specific services [3].

To our knowledge, only three studies have assessed the cost-effectiveness of interventions within rehabilitative eye care, and none of these investigated ICT training. Eklund et al. [4] investigated whether a health education program provided by community-based occupational therapists to small groups of people with age-related macular degeneration would be cost-effective compared to individually tailored programs (the usual type of care). The primary outcome measure was perceived security in performing daily activities. The results indicated that the small-group health education program was cost-effective compared to individually tailored programs. In the US, Stropue et al. [5] compared an outpatient program with a residential patient program for visually impaired veterans and found that the costs and effects (in terms of functional visual ability) had increased for both groups four months after the end of the rehabilitative care period. However, the residential patient program was more costly, and after adjusting for the baseline characteristics, the residential program was also more effective. To our knowledge, the most recent study investigating the cost-effectiveness of interventions within rehabilitative eye care was conducted by Bray et al. [6]. Compared to the previously mentioned two studies, this study compared electronic vision enhancement systems with optical low vision aids. Bray et al. [6] concluded that the electronic vision enhancement systems may be a cost-effective mean of improving near vision visual function. However, their results could not be proven cost-effective when using generic utility instruments (EQ-5D) and capability measurements (ICECAP-A).

For people with visual impairments, rehabilitative eye care services such as ICT training, can be seen as a means to increase independence and enable participation in society [2]. However, in the context of continuously rising healthcare expenditures, it is essential to optimally allocate healthcare resources. To date, there has been a limited amount of studies, which estimate the cost-effectiveness of interventions for visually impaired and no evidence of the cost-effectiveness of ICT training among visually impaired. Therefore, the aim of the present study was to assess the cost-effectiveness of ICT training for the visually impaired, as offered by two large rehabilitative eye care providers in the Netherlands, by comparing the situation before and after receiving ICT training.

## Methods

The study was carried out among visually impaired clients of two large rehabilitative eye care providers in the Netherlands who were enrolled in ICT training between July 2014 and January 2015. Annually, about 180 people received ICT training, not simultaneously with other training. Enrollees were eligible and invited by the trainers to participate if they were not receiving any other training that could bias the outcome. The ICT training included computer training (e.g., use of Word, the Internet and email) and training sessions on the use of iPhones, iPads and digital assistant devices. Because the training was tailored to each individual’s needs (differing in the length of training), no other equivalent training was available and the waiting time for being enrolled in the training was short, the study compared each enrollee’s outcomes before and after receiving ICT training. Furthermore, as the ICT training was a part of standard rehabilitative care, the use of a control group without such training was considered impossible and unethical. The outcomes were also re-measured three months after the end of the training to investigate whether the effect of the training persisted.

**Fig. 1:**
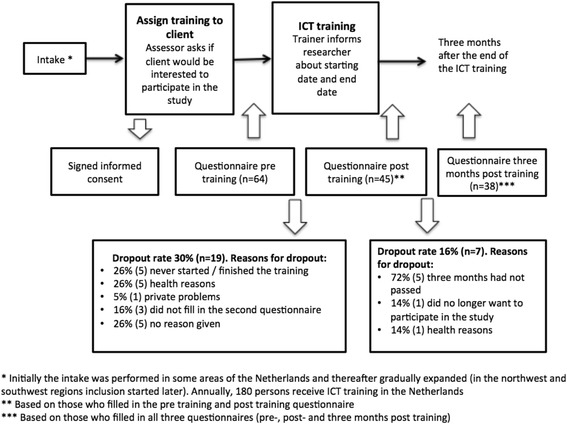
Flowchart of enrollment process, participant dropouts and time points for the questionnaire

Recruitment took place as follows. Those who were interested in the ICT training were assigned to an assessor who performed the intake and judged whether the ICT training would be feasible and appropriate. During the intake, enrollees were asked if they were willing to participate in the study. Those willing to participate then gave their informed consent. After recruitment, participants completed the first questionnaire (questionnaire pre training). Based on the intake, a revalidation plan consisting of an estimation of the scope, content and length of the training was set up by the assessor. Immediately after the last training session, enrollees were asked to fill in the second questionnaire (questionnaire post training). The last questionnaire was completed three months after the end of the training (questionnaire three months post training). The questionnaires were sent to participants by mail or digitally. In some cases when enrollees struggled filling in the questionnaire, and did not have anyone who could help them with filling in the questionnaire, researchers filled out the questionnaire together with the enrollees via telephone. See Fig. 1 for the enrollment process, dropout rates and the time points for questionnaires.

At each time point (pre training, post training and three months post training), participants were asked to fill in the same questionnaire, the pre training questionnaire additionally asked participants about their highest completed level of education and their main daily activity. The questionnaire used six different instruments, each presented in the exact same order at each time point. The respondents received the questionnaire in Dutch.

The first instruments of the questionnaire included standardized questions for measuring health-related quality of life and well-being, the EQ-5D (5-level version) and the ICECAP-O. The EQ-5D (a generic health related quality of life instrument) that comprises of five health dimensions (mobility, self-care, activity, pain/discomfort and anxiety/depression) and produces ‘utilities’ that can be used to calculate quality-adjusted life-years (QALYs) [7]. Similarly, the ICECAP-O consists of five attributes (attachment, security, role, enjoyment and control), one question per dimension and four answering categories per question. The ICECAP-O measures ‘years of full capability’, based on attributes of well-being that have been found to be important for the elderly [8]. The ICECAP-O produces a weighted index for capability, with a score of one representing full capability and a score of zero no capability. As this capability index is defined by an individual’s well-being [8], we will in the following sections present the ICECAP-O outcomes in terms of ‘years of well-being’, where a score of one represents the best possible well-being and a score of zero the worst possible well-being. During discussions with the ICT trainers about the practical concerns of the process of gathering the data, we were informed that the majority of the participants receiving ICT training were elderly (above the age of 60). Therefore, the explicit decision was made to use the ICECAP-O, as it is specifically aimed at measuring the well-being among elderly.

The third and fourth instruments used in the questionnaire comprised the Medical Consumption Questionnaire (iMCQ) [9] and the Productivity Cost Questionnaire (iPCQ). The iMCQ was used to determine respondents’ healthcare consumption during the previous two or three months, depending on the length of their training. Those who had received training for three months or longer were asked about their healthcare consumption during the preceding three months, while those who had received training for less than three months were asked about their healthcare consumption during the past two months, which was then extrapolated to three months. The iPCQ [10], which encompasses questions related to presenteeism at or absenteeism from paid work and productivity losses due to unpaid work, was used to determine productivity losses.

The final two instruments of the questionnaire were the Care-related Quality of Life (CarerQol-7D, hereafter referred to as CarerQol) and an adapted version of the Dutch Activity Inventory (D-AI) [11, 12]. The D-AI measures rehabilitation needs of visually impaired persons and rehabilitation outcomes, hence addressing the ICT needs as measured by the D-AI, is the vehicle towards possible improvement in well-being (ICECAP-O) and health-related quality of life (EQ-5D). The adapted version of the D-AI that was used included only those items relevant for evaluating the effect of the training on enrollees’ ICT skills. This resulted in 14 questions with six answering categories for each question (easy, fairly easy, difficult, very difficult, impossible, not applicable). To obtain an overall picture of ICT skills, a sum score was calculated for all 14 items (possible range 0–56). The CarerQol was used to assess the potential effect of ICT training on informal caregivers, as it is a standardized instrument for measuring and valuing the impact of providing informal care. Hence, informal caregivers were asked to complete the last part of the questionnaire.

Reference prices from the Dutch costing manual [13] were used to calculate the costs for productivity losses and medical consumption. Information regarding the total costs of the training sessions (including overheads) was provided by the rehabilitation centers.

To examine whether the outcomes before and immediately after the training differed, a t-test was conducted, as health outcomes are generally distributed normally. However, we also performed a Wilcoxon signed-rank test to validate the results. In addition, we conducted a multiple regression to investigate whether the number of training sessions, the participants’ gender or their age influenced the D-AI sum score. The cost-effectiveness analysis was performed comparing the outcomes and costs pre training and post training (*n* = 45). Four different approaches were used to calculate the incremental cost-effectiveness ratios (ICERs). The first and second were the incremental costs per QALY gained and the incremental costs per well-being year gained. The ‘utilities’ derived from the EQ-5D were used to calculate the QALYs, and the ‘utilities’ derived from ICECAP-O were used to calculate costs per ‘years of well-being’. Furthermore, we used two types of costs: the costs for ICT training alone and the combined total medical costs and costs for ICT training. Applying bootstrapping, 5000 replications were generated for the outcomes and costs before and after the training. For each replication, an ICER was calculated.

## Results

We limit the results to those respondents who filled in the pre training questionnaire and post training questionnaire (*n* = 45). This number is limited as some ICT trainers, although informed about the study, had to be reminded repeatedly to include clients. Respondents who had not completed the ICT training within the study period or who dropped out because of other reasons were excluded from the analysis (the dropout rates and reason for drop out can be found in Fig. 1). Among the respondents who filled in all three questionnaires (*n* = 38), an additional analysis was conducted to determine whether the effects of ICT training were persistent.

### ICT training costs

The mean cost of the ICT training per participant who had filled in the pre- and post-questionnaire was € 3011, and the average number of sessions per participant was 20 (range 2–63). Respondents with a higher level of education (university- or applied sciences degree) had on average slightly fewer ICT training sessions (on average 17 sessions) compared to those with a lower education level. The average cost (including overheads) per hour was € 129. In an additional scenario-analysis, we also included the costs generated by those who dropped out. The 14 respondents, who started training but dropped out, had on average 9.42 sessions. The average cost per session was € 150. In total, these sessions cost € 19,800. Distributing these costs among the 45 respondents who completed the training, gives an additional cost of (€ 19,800 / 45=) € 440 per participant. As a result, in the scenario analysis, the mean costs of the ICT training per participant became (€ 3011 + € 440 =) € 3451. Unfortunately, we do not know whether these 14 persons had benefits of their (shorter) ICT-training.

### Baseline characteristics

Of the 45 participants who completed the questionnaire pre training and post training, 58% (26) were women; see Table 1. The mean age was 63 years (range 27–90 years), and the mean quality of life score (EQ-5D) was 0.70 (range 0.1–1.0). Participants experienced trouble mainly with respect to daily activities, pain/discomfort and mobility; the percentages of participants with at least moderate problems in terms of daily activities, pain and mobility were 29%, 36% and 42%, respectively. Turning to well-being, the mean score for the ICECAP-O was 0.77 (range 0.4–1.0), and participants experienced the most restrictedness with respect to the ‘control’ domain (a low level of perceived independence): 58% felt that they were independent in only a few things or were completely dependent on others. Twenty-seven participants reported having an informal caregiver, and their mean CarerQol-score was 84 (range 14–97). The mean D-AI sum score was 23 (out of 56), indicating that participants largely struggled with various items presented in the D-AI questionnaire. For example, 82% of the participants had difficulty viewing photographs and 82% had difficulty reading the screen. In addition, 70% experienced difficulty with using the Internet and 43% with email. Regarding medical consumption, 38% of the participants received some form of home care, mainly practical household help. Furthermore, 77% had seen their general practitioner at least once during the preceding three months. The mean medical cost per participant was € 1681 for three months, the main cost driver being home care. Out of the total sample, only 11% (5) were employed. The mean productivity losses at the baseline were € 1094 per respondent for three months, solely due to their decreased ability to undertake unpaid work.

**Table 1:** Participant characteristics and results pre- and post-training

*n* = 45	Pre training	Post training
Gender (women)^a^	57.8	
Mean age (SD) range^a^	63 (17) 27–90	
Main daily activity (%)^a^		
Employed (paid work)	11.4	
Homemaker	15.9	
Incapable of working due to sickness or disability	34.1	
Early retirement	38.6	
Highest completed education (%)^a^		
No education	6.7	
Primary school	8.9	
Secondary education	57.8	
Higher education	24.5	
Other	2.2	
EQ-5D		
Mean utility score (SD) range	0.70 (0.24) 0.1–1	0.73 (0.22) 0.1–1
Mean VAS (SD) range	71 (16) 20–100	71 (16) 30–100
*At least moderate problems (%)*		
Mobility	28.8	37.8
Self-care	4.4	4.4
Daily activities	35.5	31.1
Pain/discomfort	42.2	31.1
Anxiety/depression	15.5	11.1
ICECAP-O		
Mean (SD) range	0.77 (0.13) 0.4–1	0.81 (0.13) 0.5–1
*Outcome per item (%)*		
Little/no friendship and love	24.4	22.2
Some concern/a lot of concern about the future	33.3	22.2
Able to do a few things to feel valued/unable to do any of the things to feel valued	26.6	20
Little/no enjoyment and pleasure	24.4	17.8
Able to be independent in a few things/unable to be independent	57.8	40
D-AI		
Mean sum score (SD) range	22.98 (11.9) 6–56	13.13 (8.7) 1–36
*Difficult, very difficult or impossible (%)*		
Computer skills	64.4	21.0
Screen	81.9	62.3
Keyboard	37.8	15.5
Mouse	63.3	38.0
Hotkeys	70.2	31.4
Word processor	55.3	25.8
Photographs	82.2	52.4
Internet	70.4	29.6
E-mail	42.9	11.9
Computer games	76.2	57.1
Using ICT without pain/complaints	58.1	33.3
Braille	74.9	40.0
Speech programs	39.3	10.8
Magnification software	46.9	14.4
iMCQ/iPCQ		
Mean productivity costs per 3 months	€ 1094	€ 1086
Mean medical costs per 3 months	€ 1681	€ 1825
CarerQol	*n* = 27	*n* = 26
Mean (SD) range	84 (12.0) 44–97	82 (16.8) 29–100
Mean VAS (SD) range	7.4 (1.0) 5–9	7.6 (0.9) 5–9

^a^Respondents were only asked to report this in the pre training questionnaire

### Impact of the ICT training

The health-related quality of life measured with the EQ-5D improved slightly after the ICT training, from 0.70 to 0.73. Participants experienced less pain/discomfort and anxiety/depression and fewer problems with daily activities than before the training. However, the percentage of participants who had at least moderate problems with their mobility increased from 29% at the start of the training to 38% after the last training session. Furthermore, an increase in well-being (ICECAP-O) was observed immediately after the training, with a mean score of 0.81 compared to 0.77 before the training. The most notable improvements were seen within the domains ‘security’ (an 11% decrease in reporting some or a lot of concern about the future) and ‘control’ (an 18% decrease in reporting independence in only a few things or none at all); see Table 1. The mean D-AI score (ICT skills) decreased from the initial outcome by 9.9 points, to 13.1, indicating a positive effect on ICT skills. The most noteworthy changes were observed in the areas of computer skills, the Internet and use of hotkeys.

Twenty-six participants had an informal caregiver at the end of the training, and their mean CarerQol score was 82, largely similar to the score before the training. The iMCQ revealed that 30% of the participants received home care and 60% had visited their general practitioner at least once during the preceding two or three months. The mean medical costs per respondent were € 1825 during the last three months, with the main cost drivers being hospital admissions and home care. Productivity costs per respondent were € 1086 for three months, mainly due to the productivity losses of unpaid work. Table 1 provides a more detailed description of the outcomes before and after the training.

To investigate the differences in outcomes before (pre training) and after the training (post training), a paired t-test was conducted. The utility and VAS score of the EQ-5D and the CarerQol VAS did not differ statistically (*p* > 0.05). The improvement in well-being (measured by ICECAP-O) was, however, statistically significant (*p* < 0.03). Examining each domain of the ICECAP-O, the most substantial, although non-significant, improvements were found in ‘enjoyment’ (*p* = 0.38), ‘security’ (*p* = 0.23) and ‘control’ (*p* = 0.06). Before the training, 24% of the respondents stated that they felt ‘only little enjoyment or pleasure’ or ‘none’, and this decreased to 18% after the training. In addition, while 42% of the respondents felt they were ‘completely independent’ or ‘independent in many things’ before the training, this increased to 60% after the training.

We observed improvements in ICT skills: The mean D-AI score decreased (improved) by 10 points (*p* < 0.01), and all ICT skills except computer games and braille improved; see Table 1. To further validate these results, a Wilcoxon signed-rank test was conducted, and it yielded the same results as the t-test.

Notably, the medical costs were slightly higher post training compared to pre training, due to the hospital admissions of five participants during the ICT training period, but not at a statistically significant level (*p* = 0.69). Productivity costs remained constant and were predominantly related to unpaid work (*p* = 0.98). In addition, a regression analysis was conducted to investigate whether the number of training sessions, participants’ gender or age influenced the D-AI sum score. The analysis showed that an extra training session leads to a 0.15 decrease in the sum score (positive effect). However, this was not statistically significant, and the model had limited explanatory power. The mean CarerQol score after the training was slightly lower than before the training, but this difference was not statistically significant (*p* = 0.28). Table 2 shows the outcomes before and after the training.

**Table 2:** Outcomes pre-, post-, and three months post- training

Outcomes pre-, post-, and three months post-training							
	Pre training (mean)	Post training (mean)	Three months post training (mean)	*p*-value (pre- and post-training)	95% CI differences (pre-and post-training)	*p*-value (pre- and post-training)	95% CI differences (pre- and post-training)
ICECAP-O	0.77	0.81	0.81	0.03*	0.00 - 0.08	0.91	−0.03 - 0.04
EQ-5D	0.70	0.73	0.75	0.36	− 0.03 - 0.08	0.45	−0.04 - 0.08
EQ-5D VAS	71.02	71.26	70.68	0.92	−4.84 - 5.33	0.80	− 04.94 - 3.84
D-AI sum score	22.98	13.13	12.97	0.01*	−6.51 - -13.17	0.81	−3.48 - 2.74
CarerQol^a^	83.07	81.81	77.02	0.69	−7.65 - 5.13	0.01*	− 10.96 - -1.58
CarerQol VAS	7.40	7.61	6.97	0.28	−0.16 - 0.54	0.03*	− 1.13 - -0.49

*p < 0.05

^a^The t-test for CarerQol is based on caregivers who filled in the questionnaire pre- and post-training, *n* = 24

### Does the effect of ICT training persist?

Three months after the last training session, respondents were asked to fill in the last questionnaire. The sample consisted of 38 participants, among whom 17 had an informal caregiver. A t-test comparing the scores immediately following the training (post training) and the scores three months later (three months post training) was conducted to analyze whether the effects of the ICT training were persistent. The mean D-AI score remained 13 after three months, indicating a persistent improvement in ICT skills. The mean utility score for EQ-5D appeared to be slightly higher after three months, but this was not statistically significant. The mean score for measuring well-being remained constant. The t-test revealed a significant increase in the mean CarerQol (*p* = 0.01) and CarerQol VAS (*p* = 0.03). Table 2 shows the outcomes.

### Cost-effectiveness

Because medical costs were slightly (but not significantly) higher post training (€ 1825) compared to pre training (€ 1682), we could not rule out that higher medical costs after the training may have had an impact on participants’ health-related quality of life and/or well-being. Therefore, we decided to include medical costs in the calculations.

As ICT training was shown to have persistent positive effects on health and well-being after the last training session, and the mean age among the participants was 63 years, which meant that their remaining life expectancy was 18 years [14], we assumed that the health and well-being effects would remain constant for 10 years. Because this assumption of 10 years is uncertain, we also calculated the ICERs for five and 15 years.

The results show that when only the costs for ICT training are included, the incremental costs were € 11,362 per QALY and € 7821 per well-being year gained. In contrast, when both the costs for ICT training and medical costs are considered, the costs were € 16,785 per QALY gained and € 11,553 per well-being year gained. If we assume that the effect persists for five years, the cost-effectiveness (including only the costs for ICT training) changes to € 22,725 per QALY and € 15,642 per well-being year gained. If we assume that the effect lasts for 15 years, the result is € 7575 per QALY gained and € 5214 per well-being year gained. As scenario-analysis, we also included the estimates for costs and cost-effectiveness including training costs of dropouts, assuming that the training produced no beneficial effect for the dropouts. This results in a limited increase in cost per QALY (and per year of well-being) gained. Table 3 provides an overview of the cost-effectiveness outcomes.

**Table 3:** Costs, effects and cost-effectiveness of ICT training per respondent who completed training

	QALYs gained	Well-being years gained	Differences in costs	Costs per extra QALY	Costs per extra year of well-being gained
Costs of training	0.265	0.385	€ 3011	€ 11,362	€ 7821
Cost of training (incl. costs of dropouts)	0.265	0.385	€ 3451	€ 13,023	€ 8964
Training and medical costs	0.265	0.385	€ 4448	€ 16,785	€ 11,553
Costs for training and 5-year persistent effects	0.133	0.193	€ 3011	€ 22,725	€ 15,642
Costs for training and 15-year persistent effects	0.398	0.578	€ 3011	€ 7575	€ 5214

### Uncertainty analysis

**Fig. 2:**
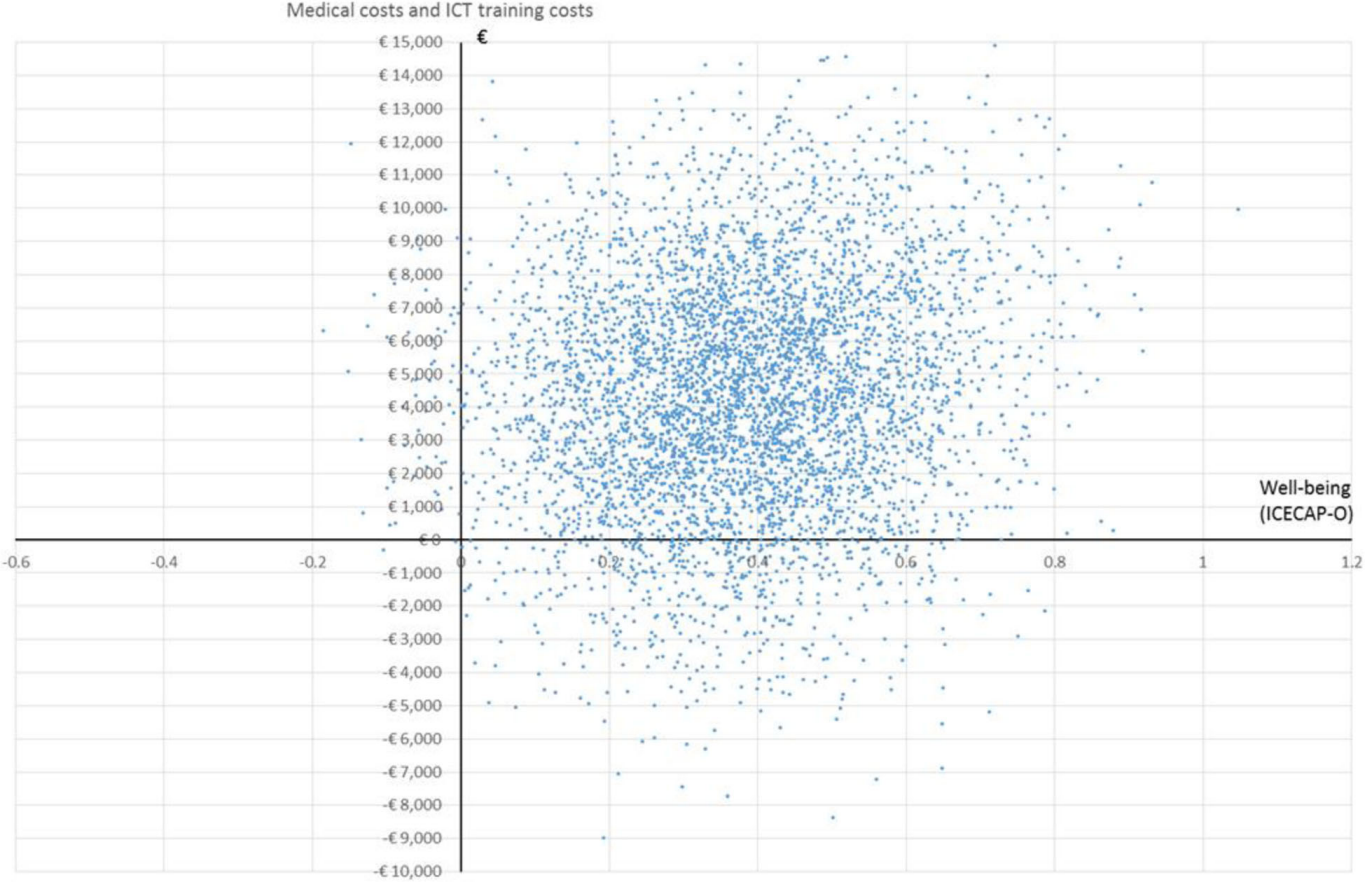
Cost-effectiveness plane (well-being, total medical costs and costs of ICT training)

Figure 2 illustrates the cost-effectiveness plane for the replicated ICERs, based on total medical costs and costs for the ICT training per gained year of well-being. The plane shows that 89% of the replicates were plotted in the northeast quadrant, indicating an increase in costs and effects. However, 1% of the replicates were plotted in the southwest quadrant, indicating a 1% chance of negative effects and increased costs. Lower costs as well as favorable effects were observed in 10% of the replicates. This is depicted in the southeast quadrant.

**Fig. 3:**
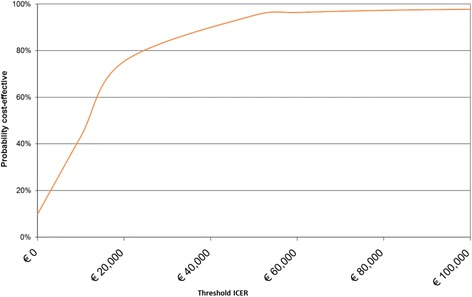
Acceptability curve for well-being and medical and ICT training costs

In addition, Fig. 3 presents an acceptability curve with different thresholds. When the willingness to pay is € 20,000 per year of well-being, then the probability that ICT training will be cost-effective is 75% (73% when including the training costs of the dropouts). With a threshold of € 50,000 per year of well-being, the probability that the ICT training is cost-effective is 95%.

When only the costs of ICT training were included (excluding the change in medical costs), the results of the bootstrapping analysis indicated that in 99% of the cases, well-being was gained with limited additional costs.

## Discussion

In the context of increasing healthcare expenditures, optimal allocation of healthcare resources is essential. To ensure future availability of services in rehabilitative eye care, it is important to investigate the costs and effects of such services. However, recent cost-effectiveness studies for rehabilitative eye care are lacking. Therefore, the aim of this study was to assess the cost-effectiveness of rehabilitative eye care with respect to ICT training for the visually impaired offered by two large rehabilitative eye care providers in the Netherlands.

The results of our cost-effectiveness study indicate that ICT training among the visually impaired has positive effects on the participants’ ICT skills and well-being. These effects also seem to persist at least three months after the last training session. Given the quality of life score of 0.7 for the respondents, their disease severity is 0.15 in terms of proportional shortfall (i.e. equity weighting, combining QALY loss with the remaining QALY expectations in the absence of the disease [15]), which implies that the willingness to pay for a year of well-being or QALY will be at most € 20,000 in the Netherlands [16]. This indicates that ICT training for the visually impaired is cost-effective as per Dutch standards, under the assumption that the effects of ICT training are persistent for 10 years.

As previously mentioned, three cost-effectiveness studies have been conducted in the area of rehabilitative eye care. Two of these studies are rather outdated, and do not use generic health technology assessment (HTA) instruments nor do they present ICERs, which makes direct comparisons with other rehabilitative (eye) care programs impossible. The third study by Bray et al. [6] is more recent and up to HTA quality standards. It showed a quite small increase in QALYS and well-being (capability) years of electronic vision enhancement systems in comparison with optical magnifiers, for people with a visual impairment. The nature of vision enhancement systems is quite different from the rehabilitative intervention evaluated in this study.

We used several generic HTA instruments (ICECAP-O, EQ-5D, CarerQol) along with a program-targeted outcome (D-AI). Our study suggests that ICECAP-O and EQ-5D have different levels of sensitivity. The overall EQ-5D score did not statistically change from before the training to after (although the underlying domains ‘mobility’ and ‘pain/discomfort’ showed some changes), while the respondents’ ICT skills increased. However, the 0.04 increase in the ICECAP-O score was relevant and statistically significant. The relevance of the 0.04 gain in well-being (capability) can be illustrated by Flynn et al. [17], analyzing 622 older people in Britain. The difference in well-being between ‘good’ and ‘fairly good’ general health was estimated as 0.029, between ‘fairly good sleep quality’ and ‘very good sleep quality’ was 0.028. Both differences appear relevant in terms of health, but are still smaller than the well-being increase as found in our study. However, further investigations should be conducted to determine the sensitivity of generic HTA instruments in the area of rehabilitative eye care. We therefore recommend that generic HTA instruments along with program-targeted instruments should be applied when determining the cost-effectiveness of rehabilitative eye care. In addition, we also experienced a very limited amount of missing values among the generic HTA instruments, which indicates that these instruments are practically applicable in the area of rehabilitative eye care.

This is the first study to use the ICECAP-O in connection with rehabilitative care for the visually impaired; Bray et al. [6] used the ICECAP-A that measures capabilities for non-elderly. We suggest that the ICECAP-O should be used more often within this type of care and that its validity and applicability should be established. Deriving well-being scores from the ICECAP-O can be considered particularly suitable for this cost-effectiveness study, as ICT training can be perceived to have a broader impact on well-being [8]. Furthermore, our results, like those of other studies that have investigated the effectiveness of rehabilitative care for people with visual impairments, show little evidence of improvement in the generic health-related quality of life [18]. In today’s society, where ICT skills are essential for full societal participation, ICT skills can be seen as a crucial determinant that can hinder societal exclusion [2] and hence affect a person’s well-being. Therefore, measuring only the health-related quality of life would likely underestimate the effects of rehabilitative eye care with respect to ICT training.

Regarding productivity costs, only five of the respondents held a paid job (11%), and only one of these reported absenteeism. This respondent was on sick leave for the entire training period but did not show any deterioration in EQ-5D, ICECAP-O or D-AI scores pre training and post training. Therefore, we excluded these productivity costs, as we did not expect in this case that productivity costs would have an impact on quality of life and well-being.

However, this study has some limitations. First, the sample size was rather small, and the results should therefore be interpreted with caution. It is advisable that economic evaluations of rehabilitative eye care should be conducted on a larger sample. Second, no control group was available, nor was randomization possible. ICT training is not a new intervention but rather a part of standard care; hence, a control group without ICT training would have been unethical. As such, this study should be seen as a ‘pragmatic trial’ (opposed to a ‘strict clinical trial’), with the aim of estimating the cost-effectiveness of the current practice with all its flaws, providing more external validity. Therefore, for example, strict registration of those who rejected to participate in the study during the intake was not performed. Third, this study did not investigate the long-term effects of ICT training, although our results show persistent effects after three months. In the cost-effectiveness analysis, we assumed that the positive effects of ICT training would persist for 10 years. However, it remains unclear exactly how long the positive effects of rehabilitative eye care interventions will persist [18]. To address the uncertainty regarding persistence, we included a more conservative assumption of five years in our cost-effectiveness analysis, which still showed to be cost-effective. However, as mentioned above, we strongly recommend further empirical research to investigate the long-term effects of ICT training in a larger group of people with visual impairments. As technology is constantly evolving, ICT requires users to keep up with technological advances. Hence, we also suggest evaluating the (cost-) effectiveness of short refresher courses in ICT training.

## Conclusion

This study suggests that ICT training among the visually impaired has positive effects on well-being and ICT skills, which also seem to persist five months after the last training session. As the respondents in our study have limited disease severity, the willingness to pay for the training in the Netherlands will be at most € 20,000 for a year of well-being or QALY. Consequently, ICT training appears to be cost-effective under the assumption that the effects of ICT training on well-being remain constant for five or 10 years. However, further research involving a larger sample and incorporating long-term effects should be conducted. As this is, to our knowledge, one of the first cost-effectiveness studies in the area of rehabilitative eye care, we hope that this study sets the scene for future economic evaluation of rehabilitative eye care.

## Abbreviations

CEA: Cost-effectiveness analysis
D-AI: Dutch Activity Inventory
HTA: Health technology assessments
ICER: Incremental cost-effectiveness ratio
ICT: Information and communication technology
iMCQ: iMTA Medical Consumption Questionnaire
iPCQ: iMTA Productivity Cost Questionnaire
QALY: Quality adjusted life-years
